# A Case of Type I Debranching Complicated by Anastomotic Pseudoaneurysm: Do Not Ask Too Much of the Ascending Aorta

**DOI:** 10.1055/s-0039-1688439

**Published:** 2019-09-17

**Authors:** Davide Carino, Alberto Molardi, Tiziano Gherli, Francesco Nicolini, Andrea Agostinelli

**Affiliations:** 1 Department of Cardiac Surgery, Parma General Hospital, Parma, Italy

**Keywords:** hybrid arch repair, TEVAR

## Abstract

Treatment of aortic arch aneurysm with standard open surgery is technically demanding, and associated morbidity and mortality are not insignificant. In high-risk patients, hybrid procedures with debranching and reimplantation or bypass of the aortic arch vessel followed by thoracic endovascular aortic repair (TEVAR) in the aortic arch represent a valid alternative to open surgery. However, when the ascending aorta is mildly dilated, the risk of retrograde dissection increases sharply. Here, we report a case of thoracic aortic aneurysm, with normal ascending aorta diameter, treated with Type I debranching and anterograde TEVAR complicated by anastomotic pseudoaneurysm and acute endocarditis, treated ultimately with ascending aortic repair and aortic valve replacement.

## Introduction

Treatment of aortic arch aneurysm with hybrid procedures has been introduced as an alternative to standard surgical repair in high-risk patients. These procedures consist of debranching and reimplantation or bypass of the aortic arch vessels followed by thoracic endovascular aortic repair (TEVAR) in the aortic arch. When the aneurysm does not involve the ascending aorta, a Type I debranching can be performed. 1 In such cases, reimplantation of the aortic arch vessels using a four- or three-branched Dacron graft sewn to the native ascending aorta just above the sinotubular junction is accomplished. The native ascending aorta in zone 0 is then used for a proximal landing zone for the stent graft. The use of cardiopulmonary bypass (CPB) in such procedures is not mandatory. However, when the diameter of the ascending aorta is greater than 3.7 cm, a Type II procedure, replacing the ascending aorta to create a suitable proximal landing zone for the stent graft before the debranching of the arch vessels, is advisable to avoid retrograde dissection and anastomotic pseudoaneurysm in the native ascending aorta. 1 Here, we report a case of thoracic aortic aneurysm treated with Type I debranching complicated by anastomotic pseudoaneurysm and acute endocarditis of the aortic valve, ultimately successfully treated with ascending aortic and aortic valve replacement.

## Case Presentation

**Fig. 1:**
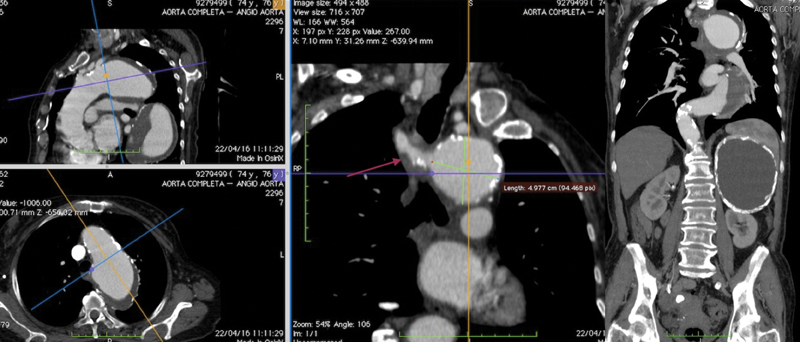
Computed tomographic angiogram showing the thoracic aortic aneurysm. Note the presence of the Kommerell diverticulum with the right aberrant subclavian artery and the huge cystic lesion of the left kidney.

A 76-year-old woman was referred to our institution after the incidental diagnosis of thoracic aortic aneurysm. Her past medical history included hypertension, myocardial infarction treated with medical therapy in the 1980s, and diverticulosis of the sigmoid colon. A transthoracic echo showed normal ejection fraction with competent valve. Her past surgical history was remarkable for cholecystectomy and appendectomy. The transverse diameters of the aneurysm were 3.5 cm in the ascending aorta, 8.2 cm at the arch, and 8 cm in the proximal descending thoracic aorta measured on computed tomographic angiogram (CTA) ( Fig. 1 ). The transverse diameter of the aorta 4 cm above the celiac axis was 3.1 cm. An aberrant right subclavian artery arising from a Kommerell diverticulum was present as well. A huge cystic lesion of the left kidney was finally noted.

Coronary angiography showed a critical stenosis of the distal circumflex artery. Echocardiography showed normal ventricular function and no significant valvular diseases.

CTA of the intracranial and supra-aortic vessels highlighted a normal circle of Willis bilateral by normal vertebral arteries.

Considering the age and the frailty of the patient, the presence of a coronary stenosis, and the normal diameter of the ascending aorta, to avoid the CPB we decided to perform Type I debranching followed by anterograde TEVAR. Considering the good caliber of the right vertebral artery, we decided to simply overstent the right aberrant subclavian artery without a carotid–subclavian bypass.

In the preoperative period, the patient developed a paroxysm of third-degree atrioventricular block and permanent pacemaker was implanted. At surgery, the ascending aorta and arch vessels were exposed through a median sternotomy. By means of a 16/10/8/8 mm Vascutek quadrifurcated graft (Vascutek Terumo, Ann Arbor, MI), the right and left carotid artery and the left subclavian arteries were rerouted to the proximal part of the ascending aorta. Three Gore C-Tag (W.L. Gore & Associates, Inc, Flagstaff, AZ) stent grafts, respectively 34 × 15, 37 × 20, and 40 × 20, were then deployed in an anterograde fashion though the fourth branch of the vascular graft using the native ascending aorta as the proximal landing zone.

**Fig. 2:**
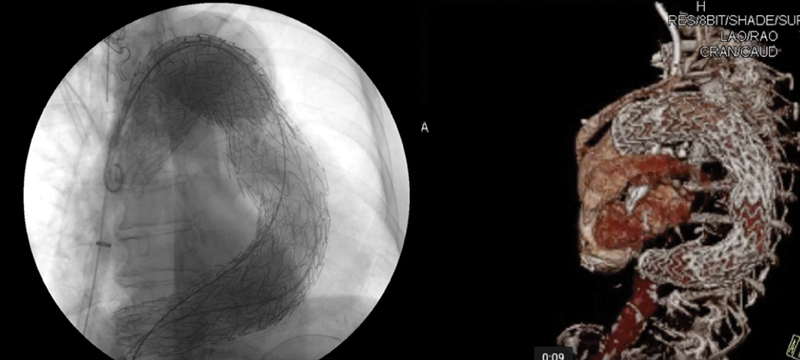
The left panel shows the intraoperative angiography with total exclusion of the aneurysm and no sign of endoleak. The right panel shows the predismissed computed tomographic angiogram with normal ascending aorta and no sign of endoleak.

Intraoperative angiography confirmed the exclusion of aneurysm and the absence of endoleak ( Fig. 2 ). Intraoperative transesophageal echo showed a normal ascending aorta. Normal flow in the right brachial artery was highlighted with a Doppler examination in the operating room at the end of the procedure.

The postoperative course was uneventful and the patient was transferred to a rehabilitation facility in good condition on the sixth postoperative day. CTA before discharge confirmed exclusion of the aneurysm with no sign of endoleak and a normal ascending aorta ( Fig. 2 ).

Three months later, the patient developed high fever with dehiscence of the subclavicular pacemaker wound with wound cultures positive for *Citrobacter* *koseri* . The wire and the battery of the pacemaker were removed and culture of the tip of the wire confirmed positivity for *C. koseri* . Despite targeted antibiotic therapy, the fever remained high, but transthoracic echocardiogram did not show signs of endocarditis.

**Fig. 3:**
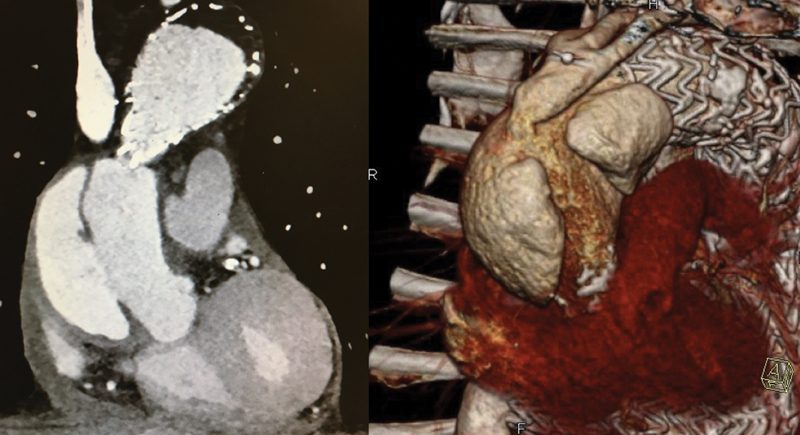
Computed tomographic angiogram showing the presence of two lumens in the ascending aorta with the three-dimensional (3D) reconstruction.

CTA was performed and showed a substantial enlargement of the ascending aorta (diameter of 6.2 cm) with the presence of two lumens ( Fig. 3 ). The patient had always been asymptomatic for chest pain. The patient was then returned to our department.

A new transthoracic echocardiogram showed a small vegetation on the aortic valve with moderate aortic regurgitation.

Surgery was then scheduled to replace the aortic valve and repair the ascending aorta. The left axillary artery and the right femoral vein were isolated and after the anastomosis of a side branch to the axillary artery, CPB was started with cooling to 24°C. A redo sternotomy was performed and the ascending aorta and the quadrifurcate graft were dissected free. The ascending aorta was cross-clamped and opened, disclosing a partially thrombosed pseudoaneurysm arising from the anastomosis of the vascular graft. The distal part of the ascending aorta appeared normal. Cold crystalloid cardioplegia was administrated in the coronary ostia. Moderate hypothermic circulatory arrest was instituted and the aortic cross-clamp was removed. The quadriforcate prosthesis was clamped at the base and bilateral anterograde cerebral perfusion was started by means of the cannula in the left axillary artery, perfusing in this way both the carotid arteries. A 32-mm Gelwave (Vascutek Terumo, Inchinnan, UK) vascular prosthesis was then anastomosed at the distal ascending aorta 1 cm before the stent graft. The quadrifurcated prosthesis was then anastomosed to the aortic graft. The quadriforcated graft was unclamped and, after dearing, the main aortic graft was clamped to start reperfusion and rewarming. The aortic valve, with a vegetation on the noncoronary sinus, was removed and replaced with a bioprosthesis (SJM Trifecta N° 21 [St. Jude Medical Inc, St. Paul, MN]). The postoperative course was complicated by acute ischemia of the left leg treated with embolectomy of the common femoral artery using a Fogarty catheter and by a major stroke. The patient was transferred to a rehabilitation facility on the 30th postoperative day.

## Discussion

Treatment of the aortic arch remains a challenge. Open repair requires CBP with hypothermic circulatory arrest, and the associated morbidity and mortality are not insignificant. 2 At the same time, the patient population with thoracic aortic disease seeking intervention gets older and older, and many patients are deemed unfit for open repair. The first description of hybrid aortic arch repair was made in 2003. 3 Since then, these procedures are being performed with increasing frequency. Moreover, the device for total endovascular aortic arch repair is now available. 4 However, the use of zone 0 for a proximal landing zone remains a concern 5 because of the high risk of retrograde Type A dissection and anastomotic pseudoaneurysm potentially related to a compliance mismatch between the stent graft and the ascending aorta. 6 Moreover, the morphological changes in the thoracic aorta induced by the cardiac cycle are quite different in the ascending aorta compared with the descending thoracic aorta. 7 Recently, it has been demonstrated that the placement of stent graft in the descending thoracic aorta straightens the ascending aorta and decreases the mean curvature of the aortic arch 8 (while the morphological changes in the ascending aorta are largely not known). However, it is reasonable to imagine that the stent graft stiffens the ascending aorta, altering meaningfully the physiological changes during the cardiac cycle. Finally, also the force exercised on the ascending aorta during the tangential cross-clamp is not negligible. To decrease the risk of retrograde dissection in patients deemed unfit for open repair or hybrid Type II repair (both of which require CPB and a variable period of circulatory arrest), when the ascending aorta diameter exceeds 4.0 cm, the use of aortic wrapping before the insertion of the stent graft has been proposed. 9 10

In our patient, although the diameter of the ascending aorta was 3.5 cm, a value that is usually considered safe to perform a Type I debranching, 1 an anastomotic pseudoaneurysm developed 3 months after the procedure. Another peculiarity of this case is the coexistence of anastomotic pseudoaneurysm and acute endocarditis. The contemporary presence of two severe conditions like these is quite rare; however, in our case two clear predisposing factors for both diseases were present: the ascending anastomosis and the infected pacemaker.

In conclusion, although hybrid Type I arch repair is an effective option, the risk of retrograde dissection and anastomotic pseudoaneurysm is a matter of concern. The radial force exercised by the stent graft together with the presence of the proximal anastomosis alter dramatically the physical characteristics of the ascending aorta, increasing the risk of retrograde dissection even at small ascending diameter. Hybrid arch repair should be tailored to every patient, reserving the Type I procedure for patients deemed unfit for CPB and Type II procedures, especially when the ascending aorta is mildly dilated.

## References

[1] Bavaria J Vallabhajosyula P Moeller P Szeto W Desai N Pochettino A Hybrid approaches in the treatment of aortic arch aneurysms: postoperative and midterm outcomes J Thorac Cardiovasc Surg 2013 145(3, Suppl):S85 S90 23260461 10.1016/j.jtcvs.2012.11.044

[2] Hiraoka A Chikazawa G Totsugawa T Objective analysis of midterm outcomes of conventional and hybrid aortic arch repair by propensity-score matching J Thorac Cardiovasc Surg 2017 154 01 100 1060 28314530 10.1016/j.jtcvs.2016.12.060

[3] Czerny M Fleck T Zimpfer D Combined repair of an aortic arch aneurysm by sequential transposition of the supra-aortic branches and endovascular stent-graft placement J Thorac Cardiovasc Surg 2003 126 03 916 918 14502197 10.1016/s0022-5223(03)00222-8

[4] Makaloski V Tsilimparis N Rohlffs F Heidemann F Debus E S Kölbel T Endovascular total arch replacement techniques and early results Ann Cardiothorac Surg 2018 7 03 380 388 30155417 10.21037/acs.2018.04.02 PMC6094014

[5] Benrashid E Wang H Keenan J E Evolving practice pattern changes and outcomes in the era of hybrid aortic arch repair J Vasc Surg 2016 63 02 323 331 26518097 10.1016/j.jvs.2015.09.004 PMC5140093

[6] Czerny M Weigang E Sodeck G Targeting landing zone 0 by total arch rerouting and TEVAR: midterm results of a transcontinental registry Ann Thorac Surg 2012 94 01 84 89 22595468 10.1016/j.athoracsur.2012.03.024

[7] Lasheras J C The biomechanics of arterial aneurysms Annu Rev Fluid Mech 2007 39 01 293 319

[8] Hirotsu K Suh G-Y Lee J T Dake M D Fleischmann D Cheng C P Changes in geometry and cardiac deformation of the thoracic aorta after thoracic endovascular aortic repair Ann Vasc Surg 2018 46 83 89 28887263 10.1016/j.avsg.2017.07.033

[9] Preventza O Bakaeen F G Cervera R D Coselli J S Deployment of proximal thoracic endograft in zone 0 of the ascending aorta: treatment options and early outcomes for aortic arch aneurysms in a high-risk population Eur J Cardiothorac Surg 2013 44 03 446 452, discussion 452–453 23515170 10.1093/ejcts/ezt068

[10] Gelpi G Vanelli P Mangini A Danna P Contino M Antona C Hybrid aortic arch repair procedure: reinforcement of the aorta for a safe and durable landing zone Eur J Vasc Endovasc Surg 2010 40 06 709 714 20870433 10.1016/j.ejvs.2010.08.017

